# Biomarker-guided withdrawal of inhaled corticosteroids in asthma patients with a non-T2 inflammatory phenotype – a randomized controlled trial study protocol

**DOI:** 10.1186/s12890-023-02679-y

**Published:** 2023-10-04

**Authors:** Christiane Hammershaimb Mosbech, Nina Skavlan Godtfredsen, Charlotte Suppli Ulrik, Christian Grabow Westergaard

**Affiliations:** 1 Department of Respiratory Medicine, Copenhagen University Hospital - Hvidovre, Kettegaard Allé 30, Hvidovre, Denmark; 2 Institute of Clinical Medicine, University of Copenhagen, Blegdamsvej 3B, Copenhagen, Denmark

**Keywords:** Asthma, Non-T2, ICS tapering, Randomized controlled trial, Biomarker-guided therapy, Periostin, Asthma control questionnaire

## Abstract

**Background:**

Non-T2 asthma is characterized by the absence of elevated type 2 inflammatory biomarkers such as blood-eosinophils, total and allergen-specific Immunoglobulin E and Fractional exhaled Nitric Oxide (FeNO). According to guidelines, inhaled corticosteroids (ICS) are the cornerstone of asthma management. However, ICS treatment is associated with a risk of local side effects, including hoarseness and thrush, and long-term high-dose therapy may cause systemic adverse effects. Furthermore, whereas treatment with ICS is highly effective in T2 asthma, studies have shown a markedly reduced ICS efficacy in patients with a lower degree of T2 inflammation, thus posing a clinical challenge in this subgroup of patients. Hence, owing to the ICS dosage step-up approach in current clinical guidelines, patients with low T2 biomarkers are at risk of being exposed to high doses of ICS, and by that at risk of side effects. Thus, an ICS-treatment regime guided by biomarkers that reflects the inflammatory phenotype is warranted in order to reduce the corticosteroid burden in patients with non-T2 asthma. This study combines a panel of non-T2 inflammatory markers (low periostin, low blood-eosinophils, and low FeNO), to determine if this group of patients can maintain asthma control during ICS withdrawal.

**Methods:**

This is an ongoing prospective multicenter open-label randomized, controlled trial aiming to assess if ICS can be safely tapered in patients with non-T2 asthma. The patients are randomized 1:1 to either standard of care or an ICS tapering regimen (*n* = 55 in each group) where the initial ICS dose is reduced by 50% for 8 weeks followed by total ICS removal. The primary endpoint is change in asthma control questionnaire (ACQ) from baseline to post-tapered ICS. The secondary endpoints are time from baseline to drop-out caused by loss of asthma control, changes in serum-periostin, blood-eosinophils, FeNO, Forced Expiratory Volume in 1 s (FEV1) and in sputum-eosinophils.

**Discussion:**

This study aims to provide data on ICS tapering in non-T2 asthma patients and to contribute to a more individualized and corticosteroid-sparing treatment regime in this group of patients.

**Trial registration:**

Clinicaltrials.gov Identifier: NCT03141424. Registration date: May 5^th^, 2017.

## Background

Asthma is a heterogeneous disease that can be classified into phenotypes based on inflammatory characteristics as T2 and non-T2 [1, 2]. T2 inflammation involves T-helper 2 (Th2) lymphocytes and type 2 innate lymphoid cells (ILC2) that secrete proteins such as interleukin (IL)-4, IL-5, IL-9, and IL-13. These interleukins promote recruitment of eosinophils (IL-5), basophils, and mast cells (IL-9) into the airways [2, 3].

Non-T2 asthma is poorly defined but is characterized by asthma with absence of signs of heightened T2-driven inflammation. The definition is thus traditionally based on the absence of eosinophils in sputum, blood, and bronchial mucosa and/or low values of other biomarkers of eosinophilic inflammation, such as fractional exhaled nitric oxide (FeNO) or serum-Periostin. T2 asthma also includes allergic asthma, which is triggered by a process dependent on allergen-specific immunoglobulin E (IgE). In contrast, non-T2 asthma does not involve the IgE inflammatory pathway.

The prevalence of non-T2 asthma is difficult to estimate, as many studies are cross-sectional and influenced by concomitant corticosteroid treatment and airway infections [4]. However, non-T2 asthma has been shown to be related to older age, obesity, smoking, high symptom-burden and a higher exacerbation rate, as well as increased treatment resistance to corticosteroid [5, 6].

### Previous research

According to treatment guidelines, all asthma patients should be treated with inhaled corticosteroids (ICS), regardless of inflammatory phenotype [7]. ICS treatment can cause local side effects in the airways, while long-term and high-dose use may potentially cause systemic adverse effects. The most frequently reported systemic effects were described in a systematic review and include adrenal suppression, reduced growth velocity, hyperglycaemia, diabetes, osteoporosis, respiratory infections, and cataract [8]. However, a limitation to this systematic review was the inconsistent adjustment for oral corticosteroid exposure in the included studies. Treatment with ICS are efficient in asthma, but the efficacy is reduced in patients with a lower degree of T2 inflammation [9, 10]. Considering this and the step-up algorithm of ICS dosage in asthma treatment guidelines [7], the patients with low T2 biomarkers are at high risk of being exposed to high doses of ICS, and by that an increased risk of adverse effects. Thus, a treatment regime guided by biomarkers based on the inflammatory phenotype, rather than only clinical parameters, could have a corticosteroid-sparing perspective and thus would be particularly beneficial for patients with non-T2 asthma.

Because of the need for high doses of corticosteroids in patients with severe asthma, the adverse effect profile of corticosteroids is a specific issue in these patients. Therefore, to reduce the burden of corticosteroids, non-steroid agents targeting asthmatic inflammation in severe asthma have been developed over the past decades. A panel of biological therapies have thus been approved for severe eosinophilic or allergic asthma [11–14], whereas no current biological treatment option is available to specifically target patients with non-T2 disease. Tezepelumab, a humanized monoclonal antibody targeting the upstream asthma inflammatory mediator Thymic Stromal Lymphopoietin (TSLP), is a potential treatment option for severe non-T2 asthma patients [15]. In a phase II study, tezepelumab reduced exacerbation rates by up to 71% compared to placebo independently of phenotype. Although tezepelumab is approved for treatment of severe asthma regardless of inflammatory phenotype, recent studies have shown that tezepelumab is more beneficial in T2 asthma than non-T2 asthma [16]. This may limit the advantages of tezepelumab in non-T2 asthma patients.

Another pharmacological treatment option for patients with more severe non-T2 asthma include low-dose azithromycin [17] which has been shown to reduce exacerbation rate in both T2 and non-T2-patients, though with a slight tendency towards being more effective in T2 patients. In an earlier study, however, azithromycin was shown to be efficacious in reducing exacerbations only in non-T2 asthma patients [18], rendering azithromycin as a potential treatment option for non-T2 asthma.

Evidence suggests leukotriene receptor antagonists (LTRAs) to be effective in reducing symptoms in asthma associated with allergic rhinitis, exercise-induced asthma, and aspirin-exacerbated respiratory disease [19]. No studies have investigated if there is a difference in treatment response of LTRAs in T2 and non-T2 asthma, but the above-mentioned associations point towards a more favorable response in T2 asthma.

However, all taken together, because of the limited availability of effective treatment options for non-T2 these patients may suffer a risk of being overexposed to corticosteroids. There is a need for a refinement of the treatment algorithm to include biomarkers of the inflammatory phenotype, in order to identify the patients who may not benefit from treatment with ICS. A few studies addressing this issue are available. In one study, patients were switched from ICS to long-acting β2 agonist (LABA) [20], and showed a significant risk of loss of asthma control. This study conducted in 2001, did not consider inflammatory profiles when ICS was withdrawn.

In 2006 the SMART study showed that add-on salmeterol to usual treatment with ICS for asthma reduced exacerbations and hospitalizations due to asthma, but also increased the risk of serious adverse events [21]. It is important to note that the SMART study did not take into account inflammatory phenotypes. While the study had several limitations, it highlighted the importance of balancing the benefits and potential risks of using LABA in the treatment of asthma.

In the SIENA study, the patients were divided into two inflammatory phenotypes based on sputum eosinophil count. Patients received ICS, long-acting muscarinic antagonist (LAMA), or placebo. In the low-eosinophil group, the treatment response to ICS (57%) did not differ significantly from the treatment response to LAMA (60%). In the high-eosinophil group, the ICS treatment was significantly better [22]. These results suggest that ICS may not necessarily be a mandatory component of asthma therapy, at least in milder disease.

A real-life study of the treatment effect of ICS in non-T2 asthma patients with a mixed disease severity showed that complete tapering-off ICS was possible in 39% of the patients (*n* = 14) and that the ICS dose was reduced in 28% of the patients (*n* = 10). The symptom control or exacerbation rate were not affected [23]. High blood-eosinophils at baseline or high blood- or sputum-eosinophils during the tapering were predictors for an unsuccessful tapering-of ICS. These findings indicate that ICS removal or reduction may be an option in a substantial proportion of the asthma patients, guided by the level of eosinophils.

Several biomarkers have shown some association to the T2 phenotype, including high FeNO [24], positive mannitol provocation test [25], and high sputum-eosinophils [26]. However, FeNO is not 100% specific for eosinophilic inflammation and the sensitivity is poor [27], so this test cannot stand alone. The other available tests have practical and clinical challenges, so easily applicable biomarkers for phenotyping asthma are needed.

High values of serum-periostin are associated with T2 asthma [28]. Periostin is a matricellular protein secreted by bronchial epithelial cells when exposed to IL-13 and IL-4. This biomarker plays a role in several pathogenic processes in asthma, including airway remodeling, subepithelial fibrosis, eosinophil recruitment and regulation of mucus production in goblet cells [29]. Thus, serum-periostin may be a potential easily accessible biomarker to guide ICS-treatment. The evidence in this area is very sparse. However, a recent study of severe asthmatics did compare a composite biomarker-guided vs. symptom-guided treatment with ICS. The composite biomarkers included serum-periostin, FeNO and blood eosinophils. The biomarker-guided algorithm successfully reduced the ICS dose when compared to the symptom-guided regimen in a per-protocol population [30]. The patients who reduced ICS dose did not experience loss of symptom control or change in biomarkers.

In the future management of asthma, there is a need to fully acknowledge the heterogeneity of the disease and turn towards more targeted and individualized treatments and away from a ‘one-size fits all’ strategy. Therefore, a clinically applicable method to determine the group of patients with low ICS efficacy is needed. In this study we build upon knowledge from currently available studies and apply a panel of biomarkers to phenotype asthma patients, thus identifying patients who do not express T2 inflammation. The aim of this RCT is to determine whether it is possible to withdraw ICS in this subgroup of patients without loss of symptom control. In order to test the non-inferiority of this phenotype-based treatment strategy, it is compared to standard care.

## Methods

### Objective

To investigate whether patients expressing non-T2 asthma, assessed by the biomarkers: serum-periostin, blood-eosinophils, and FeNO, can maintain their level of disease control during tapering of ICS.

### Primary endpoint

Change in Asthma Control Questionnaire (ACQ) from baseline to post-tapered ICS. Clinically significant change in ACQ will be defined as 0.5 points, and an increase in ACQ of at least 1.0 point will resolve in withdrawal from the study.

### Secondary endpoints

Time from baseline to drop-out due to worsening of asthma control.

Change from baseline in serum-periostin, blood-eosinophils, FeNO, forced exhaled volume in 1. second (FEV1) and sputum-eosinophils.

### Study design, randomization, and intervention

This trial is a randomized, controlled, multicenter, non-inferiority study to evaluate the difference between ICS tapering and usual care in patients expressing non-T2 asthma.

When all criteria are met, participants will be randomized 1:1 into the following treatment arms:

A Usual care:
  i. Patients continue their inhaler therapy in unchanged doses throughout the study period.
  ii. Patients on single maintenance and reliever therapy regime (ICS/formoterol) continue ICS/formoterol as reliver therapy.
B ICS tapering regime:
  i. Patients on ICS as monotherapy: 50% reduction of the initial dose of ICS treatment for 8 weeks, followed by ICS removal.
  ii. Patients on ICS as a combination therapy: Same procedure for ICS tapering. LABA, LAMA, LTRA, and/or theophylline will continue in unchanged dose(s). Thus, most patients will have to change to two (or three) inhalers, as ICS will have to be delivered from an ICS-only containing inhaler. ICS not available in a single-medication inhaler will be replaced with budesonide in an equipotent dose.
  iii. Patients on single maintenance and reliever therapy regime (ICS/formoterol): Short-acting β2-agonist (SABA) will replace ICS/formoterol as reliever therapy.

The allocation sequence is in blocks of varying and blinded size and generated in R 4.1.0 by an employee who is not a part of the study. The allocation list is uploaded to Research Electronic Data capture (REDcap) where randomization is performed. The trial is not blinded.

### Identification of eligible patients

**Fig. 1:**
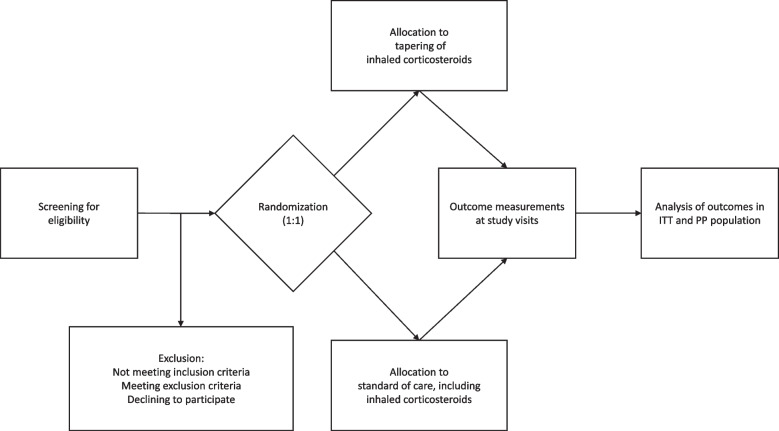
Flowchart of patients through the study. Legend: ITT: Intention-to-treat; PP: Per-protocol

Patients with asthma followed in the respiratory outpatient clinics meeting the inclusion criteria and none of the exclusion criteria, based on routine measurements, and available for assessment, will be invited to a screening for non-T2 asthma (Fig. 1)

.

After obtaining written informed consent, the screening visit will be performed, including clinical interviews, review of eligibility criteria, blood samples and measuring of FeNO. The principal and sub-investigators enroll and randomize participants and perform all study visits.

### Inclusion criteria

- Patients with asthma followed in the respiratory outpatient clinic at
  - ◦ Hvidovre Hospital, University of Copenhagen, Hvidovre, Denmark
  - ◦ Amager Hospital, Copenhagen, Denmark
  - ◦ Glostrup Hospital,  Glostrup, Denmark
- Eligible individuals are required to have at least one, at present or previously, positive asthma test:
  - ◦ FEV1 reversibility of at least 12% (and at least 200 ml) after administration of bronchodilator or inhaled/oral corticosteroid.
  - ◦ Positive bronchial provocation test, e.g., mannitol or methacholine.
  - ◦ Peakflow-variation of at least 20% over a two-week period with peak-flow measured twice daily and during asthmatic symptoms.
  - ◦ Variability in FEV1 over time of at least 12% (and at least 200 ml).
- 18 to 65 years of age.
- Treated with ICS daily in medium dose or higher, equivalent to 800 µg budesonide.
- ICS adherence of at least 80% during the last year.
- FeNO < 25 ppb at all visits at the outpatient clinic prior to the screening visit.
- Blood-eosinophils < 0,15 × 10^9^ cells/L at screening.
- Women of fertile age: negative urine-hCG and a statement of secure anticonception during the entire trial period.
- Signed informed consent.

### Exclusion criteria

- History of allergic asthma.
- Diagnosed pneumonia by a physician within the last 6 weeks before screening.
- Daily smoking or former daily smoking within the last 6 months.
- Known other respiratory conditions, including COPD and bronchiectasis.
- Known other chronic conditions that could impact or limit study participation, including severe heart disease and disorders requiring treatment with immunosuppressive drugs such as prednisolone, methotrexate, or biological therapy, as assessed by the investigator.
- Pregnancy or planning to become pregnant.
- Abuse of alcohol or other substances.

### Data management

Each participant randomized will be given a unique study ID, recorded in the electronic Case Record Form (e-CRF) together with the allocated treatment arm. The e-CRF are stored securely in REDcap, an electronic database. All study visits will be registered in the electronic patient file and the e-CRF, which will be the data sources. All data are anonymized and double-checked for errors when entered in the e-CRF in REDcap. Registrations and changes in the database are automatically logged in an audit trail. The investigators will have access to the database. The study will be monitored repeatedly by an independent monitor according to Good Clinical Practice (GCP) by the GCP unit at Bispebjerg Hospital, University of Copenhagen, Copenhagen, Denmark.

### Measurements

These will be collected as described in Table 1.

- ACQ: A validated questionnaire used for assessing the level of asthma control [31].
- FeNO: Exhalation test used for assessing eosinophil airway inflammation. The test will be performed according to ERS/ATS guidelines.
- Spirometry: A test for lung function. The test will be performed according to ERS/ATS guidelines.
- Serum-periostin: Blood sample.
- Blood-eosinophils: Blood sample (leucocytes and differential count).
- Induced sputum: Analysis including cell count of mucus collected after inhalation of hypertonic saline. The test will be performed according to standardized method [32, 33].
- Adherence: At screening adherence is measured as medical possession rate (MPR), defined as the number of doses the patient had access to divided by the total number of doses the patient was supposed to take based on their prescription obtained from the Common Medication Card, a Danish data registration of prescribed pharmaceuticals. At study visit 1 – 7 adherence is monitored on the dose counter of the inhaler.

**Table 1:** Data collected at screening, baseline, and follow-up visits

Data collected	Screening	Baseline	4 weeks	8 weeks	12 weeks	16 weeks	26 weeks	52 weeks
	Visit 0	Visit 1	Visit 2	Visit 3	Visit 4	Visit 5	Visit 6	Visit 7
ACQ		x	x	x	x	x	x	x
FeNO	x	x	x	x	x	x	x	x
Spirometry		x	x	x	x	x	x	x
Blood sample: Eosinophils	x	x	x	x	x	x	x	x
Blood sample: Periostin	x	x		x		x		x
Sputum: Differential count		x						x
Urine-hCG	x							
Adherence^a^	x	x	x	x	x	x	x	x

^a^At screening adherence is measured as medical possession rate (MPR), defined as the number of doses the patient had access to divided by the total number of doses the patient was supposed to take based on their prescription. At study visit 1 – 7 adherence is monitored on the dose counter of the inhaler

### Medication

This study will include patients treated with ICS approved for asthma treatment in Denmark, including combination treatment with LABAs and/or LAMAs. The following drugs have been defined as IMPs (investigational medicinal product) in the study (approved by the Danish Medicines Agency): Budesonide (ATC: R03BA02), fluticasone propionate (ATC: R03BA05), mometasone furoate (ATC: R03BA07), fluticasone furoate (ATC: R03AK10), beclometasone dipropionate (ATC: R03BA01), and ciclesonide (ATC: R03BA08).

The study drugs will be additionally labelled (not covering the original label) with the following information: Name of sponsor/investigator, trial reference code “PERIOSTIN”, trial site, and participant ID (randomization number).

### Safety

In case of the occurrence of one or more of the following criteria during the trial period, the participant will be withdrawn from the study at the day of the event.

- Increase in ACQ of at least 1.0 point (in total, 7 points) as compared to baseline.
- A FeNO measurement > 50 ppb that is attributed to increased asthma activity and not other factors such as infection.
- Asthma exacerbation, defined as the need for systemic corticosteroids for at least 3 days’ duration and/or hospitalization due to worsening of asthma symptoms.
- New fulfilling of an exclusion criteria during the trial period, including (pregnancy and daily smoking).
- Withdrawal of consent. In addition, participant data may be omitted after study completion upon request of the participant.

If a patient is withdrawn from the study due to a significantly increase in ACQ, FeNO and/or an asthma exacerbation, the following data will be collected at the day of the event: ACQ, FeNO, spirometry, blood-eosinophils, serum-periostin, sputum-eosinophils and adherence.

The decision of exclusion not requested by the participant will be made by the investigator. The participant will be informed.

Hereafter, the patient will attend the outpatient ward in a standard asthma course, as assessed by the study physician.

Adverse events (AE) and serious adverse events (SAE) will be registered in the e-CRF. SAEs that are suspected to be associated to the IMP are reported by the investigator to the sponsor within 24 h. An annual safety report regarding the SAEs will be conducted to the Ethics Committee of The Capital Region of Denmark and the Danish Medicines Agency.

Based on the incidence of SAEs the sponsor may conduct an interim analysis, which will be analyzed by the study group. The sponsor and investigators can in collaboration decide to terminate the study.

### Sample size and statistical considerations

#### Sample size calculation

Based on a previous study we expect it is possible to include a maximum of 50 patients in each treatment arm [23]. The primary endpoint is a change in mean ACQ from baseline to week 16 of + 0.10 points in the intervention group compared with no change in the control group. A standard deviation of 0.92, an α = 0,05, and a non-inferior margin on 0.5 points will result in a power of 70%. We anticipate a 10% drop-out rate, 110 patients in total are planned to be included in the study.

#### Descriptive data

Categorical variables will be presented as number of observations and percentages. Whereas continuous variables will be presented as mean and standard deviation or median.

#### Primary and secondary outcomes

To evaluate the effect of the intervention on the primary endpoint (change in ACQ), data will be analyzed with two-sample t-test and linear regression. Subgroup analyses on patients with different disease severity will be performed with interaction analysis in linear regression models. The variables adjusted for are BMI and previous smoking (pack years).

Secondary outcomes will be analyzed with two-sample t-test.

Time to drop-out due to worsening of symptoms or asthma exacerbation will be estimated as a hazard ratio in a Kaplan–Meier curve.

If normality assumptions cannot be met, data will be analyzed with non-parametric tests such as Wilcoxon rank test or transformed to meet the normality assumptions. Study outcomes are analyzed in the intention-to-treat and per-protocol population.

Statistical analyses are performed with R 4.1.0 (R Foundation for Statistical Computing, Vienna, Austria).

#### Publication plan

The findings from the present trial, positive, negative as well as inconclusive, will be sought published in English-language peer-reviewed journals. Results that cannot be published in peer-reviewed journals will be presented at scientific conferences as posters or oral presentations. The Vancouver declaration will be applied also regarding co-authors. CHM, NSG, CSU, and CGW can, in agreement, decide to include other co-authors.

The results are planned to be submitted for publication within 12 months after last patient last visit.

## Discussion

In this multicenter randomized controlled trial, we apply a panel of biomarkers to phenotype asthma patients, thus identifying patients who do not express T2 inflammation, in order to investigate whether ICS can be tapered in this subgroup of patients.

The true prevalence of non-T2-asthma remains uncertain. Some studies suggest that non-T2 asthma may account for up to 50% of all asthma patients [34], while others report a much lower prevalence, around 20% [35, 36]. The prevalence may vary depending on the population being studied and the methods used to identify and classify asthma subtypes. For example, non-T2 asthma may be more prevalent in certain populations such as smokers, elderly, or obese patients [35]. This uncertainty is largely due to the fact that the definition of non-T2 asthma is still evolving, and there is not yet consensus on which biomarkers best identify this form of asthma. Furthermore, the cut-off levels for T2 biomarkers may vary depending on the specific assay used and clinical context [37]. Potentially this study will contribute to finding a more accurate cut-off level of periostin for non-T2 asthma, as evidence in previous studies are conflicting [38].

Medium- to high-dose ICS are expected to reduce FeNO levels and potentially blood-eosinophils. Thus, this study is designed with frequent follow-up visits with measurements of T2 biomarkers, ensuring that suppressed T2-inflammation will be revealed during ICS tapering. A strength of the present study is that with a low threshold of non-T2 biomarkers we include patients with a good probability of success in tapering ICS. Finally, there may be limitations in the diagnostic tests used to identify non-T2 asthma, particularly in clinical settings where more advanced biomarker testing may not be available. This can lead to underdiagnosis or misclassification of patients with non-T2 asthma. The biomarkers applied in this study are characterized by being non-invasive, fast, available, relatively cheap, and easily applicable in a busy everyday clinical setting. The study has been designed to resemble a standard asthma management setting, to make the results of the trial easily transferrable to a clinical setting. To continue a pragmatic approach, it is conducted as an unblinded study.

Adherence to ICS are crucial in the management of asthma, and it has a significant impact on treatment outcomes [39]. Non-adherence to ICS has been associated with poor asthma control and increased healthcare utilization [40]. However, it could be speculated that especially patients with non-T2 asthma are even less adherent to ICS treatment owing to a perception of lack of effect. A commonly used threshold for good adherence is taking 80% or more of the prescribed doses, meaning that the patient is missing no more than 20% of the doses [39]. Furthermore, the patient needs to take the right dose, at the right time, using correct inhalation technique. A strength to this study is the inclusion of patients with > 80% adherence, as this will allow for a more accurate assessment of the efficacy and safety of the intervention being studied. The participants continue their well-known inhaler device, and we do not switch to a placebo-device, to limit loss of asthma control due to the switch of inhaler device. Furthermore, we test inhaler technique at baseline visit and assess adherence at every visit throughout the study period.

Patients in both arms go through the same follow up visits and undergo the same examinations (Table 1). When ICS has been withdrawn in the intervention arm, no changes will be made in inhaler therapy in the follow-up period of the study, so any performance bias is reduced.

In summary, the rationale of this study is that a subgroup of asthma patients expressing T2 low asthma have limited effect of the treatment with ICS. These patients will not suffer from loss of asthma control within one year after withdrawal of treatment with ICS. The aim is to investigate whether asthma patients expressing low T2-activity, assessed by serum-periostin, blood-eosinophils, and FeNO can sustain their level of disease control during tapering of ICS. The overall aim of the study is thus to reduce the burden of corticosteroids in these patients. The study will contribute to a more targeted and individualized treatment than the current one-size fits all inhalation therapy.

## Trial status

Patient recruitment commenced in June 2022 and is ongoing.

## Data Availability

Data are not yet available for this study. When the study is completed, the datasets analyzed are available from the corresponding author on reasonable request.

## Abbreviations

ACQ: Asthma Control Questionnaire;
ATC: Anatomical therapeutic chemical code
ATS: American Thoracic Society
BMI: Body Mass Index
COPD: Chronic obstructive pulmonary disease
ERS: European Respiratory Society
e-CRF: Electronic Case Report File
FeNO: Fractional exhaled nitric oxide
FEV1: Forced exhaled volume in 1 second
GCP: Good Clinical Practice
GINA: Global Initiative for Asthma
hCG: Human chorionic gonadotropin
ICS: Inhaled corticosteroids
IgE: Immunoglobulin E
IL: Interleukin
ILC2: Type 2 innate lymphoid cells
IMP: Investigational medicinal product
ITT: Intention-to-treat
LABA: Long acting β-agonist
LABD: Long-acting bronchodilators
LAMA: Long-acting muscarinic antagonist
LTRA: Leukotriene receptor antagonist
MPR: Medical Possession Rate:
Non-T2: Non-type 2
PP: Per-protocol
REDCap: Research Electronic Data Capture
SABA: Short-acting β2-agonist
SIENA: Steroids in Eosinophil Negative Asthma
SMART: Salmeterol Multicenter Asthma Research Trial
T2: Type 2
Th2: T-helper 2
TSLP: Thymic stromal lymphopoietin
